# The clinical and economic burden of rare‐earth magnet ingestion in children: A national study

**DOI:** 10.1002/jpr3.70298

**Published:** 2026-11-10

**Authors:** Lina Al‐Ani, Safaa Najar, Fajer Al‐Ishaq, Akshay Bangera, Azka Ahmad, Mareah Mancha, Hassan Baghazal, Noora Alshahwani

**Affiliations:** ^1^ Weill Cornell Medical College Ar‐Rayyan Qatar; ^2^ Pediatric Surgery Department Hamad Medical Corporation Doha Qatar; ^3^ General Surgery, Hamad Medical Corporation Doha Qatar; ^4^ General and Thoracic Surgery Department Sidra Medicine Doha Qatar

**Keywords:** costs, foreign body ingestion, neodymium magnets, pediatric, rare‐earth magnets

## Abstract

**Objectives:**

We aimed to determine the case load and morbidities of magnet ingestion‐related injuries in children of Qatar, as well as the medical costs associated.

**Methods:**

We reviewed the medical records for cases seen in Qatar from 2011 to 2024 across two tertiary care centers.

**Results:**

37/45 (82.2%) cases admitted required intervention, while 8/45 (17.7%) needed none. 20/37 (62.5%) underwent laparotomy, 4/37 (12.5%) underwent laparoscopy as the sole or combined modality, and 4/37 (12.5%) had endoscopic removal. Post‐intervention complications were reported in 9/37 cases (28.1%), including superficial wound infection (5/32), collection (3/32), adhesive bowel obstruction (1/32), and incisional hernia (1/32). No deaths were seen. In logistic regression, presence of symptoms was the only predictor for the need of intervention (odds ratio [OR] 7.09 [95% confidence interval, CI 1.23–40.75]). The total cost of care for 39 patients seen was USD 524,660.87 for a total of 229 hospital days, with a median hospital related cost per patient of USD 7901.96 (interquartile range [IQR] 3922.06, 13,699.88). Costs associated with observation only were about a fifth of that where intervention was needed (a median of USD 2056.92 per patient (IQR 1074.46, 2658.53) versus USD 10,564.37 per patient (IQR 6374.11, 14,899.4) respectively). Costs related to use of operating room and inpatient admission contributed the most, compared to medications and emergency care.

**Conclusion:**

Magnet ingestion in children in Qatar carries a significant morbidity and has significant hospital‐related costs. Policymakers should consider product recall to reduce these effects. Raising public awareness of the hazards and limiting access to strong magnets in toys would be important prevention steps.

## Abstract (highlights)

**What is Known:**

- Ingestion of rare‐earth magnets often leads to fistula formation, adhesions, obstruction, and perforation.
- Many centers have developed algorithms that involve that involve immediate intervention or intervention after a period of observation.
- The majority of magnet ingestion, require an intervention which may range from endoscopy to laparotomy.

**What is New:**

- In this study, we attempt to analyze characteristics of the intervention group compared to the group that was managed conservatively.
- The presence of symptoms is a strong predictor of intervention.
- The management of magnet ingestion‐related injuries is costly, and creates a significant burden on hospital resources.

## 1 INTRODUCTION

Rare‐earth magnet ingestion is a global health hazard for children. These special magnets were first invented by General Motors and Sumitomo Special Metals (Japan) in 1982 and have been widely used in toys, kitchen utensils, desk items, and many household products.^1^ These magnets contain neodymium, iron, and boron, which produce a strong magnetic field that is more than five times the force seen with regular magnets.^2^ Commercialized as colorful toys under the name of Buckyball and NeoCube magnets, among others, are easily accessible to children. Multiple reports have been published since the first report of morbidity following ingestion in children in 2002.^3^, ^4^

Ingestion of multiple magnets often leads to fistula formation, adhesions, obstruction, and occasionally perforation and peritonitis.^3^, ^4^, ^5^ A multicenter retrospective study in the United Kingdom's major trauma centers showed that out of 53 magnet ingestion cases seen over 1 year, 27 (50.9%) required intervention, including bowel resection and stoma.^6^ To the best of our knowledge, there has been only one reported death as far as we know.^7^ Since 2012, in recognition of the serious risk posed to children, consumer safety and regulatory authorities in Canada, Australia, and New Zealand have prohibited the sale of these products.^7^, ^8^, ^9^, ^10^

There have been no official recalls of this product in Qatar. However, since 2014, there have been multiple media campaigns to spread awareness. All cases of witnessed or suspected ingestion were referred to the pediatric surgery services. In this study, we conducted the first national‐level case series to examine the effects of rare‐earth magnet ingestion in children in Qatar, as well as quantify the hospital resource utilization, especially that related to costs. This will help inform legislation bodies of the dangers associated with these toys, the burden on healthcare and hopefully lead to preventative measures.

## 2 METHODS

### 2.1 Ethics statement

Ethical approval was secured from HMC (MRC‐01‐22‐399) and Sidra Medicine (IRB# 1903034), and all methods were performed in accordance with the guidelines approved by Ministry of Public Health (MOPH). Informed consent for included patients was waived by an Institutional Review Board (IRB).

### 2.2 Criteria for inclusion

We conducted a retrospective chart review of children aged <18 years over a 13‐year period (January 2011 to February 2024) in Qatar. All patients with suspected or witnessed magnet ingestion were referred to the pediatric surgical care service. The service was available at the Hamad Medical Center (HMC) until 2018 and was transferred to Sidra Medicine in 2018. All interventions undertaken for magnet ingestion or any other foreign body were carried out by the pediatric surgery service.

### 2.3 Data collection

Medical records were examined for demographics, clinical presentation, imaging modalities used, treatment modality (observation, endoscopic intervention, operative intervention), operative findings (if applicable), and postoperative course. The outcomes of interest were the need for operative intervention, length of hospital stay, complications observed (perforation, obstruction, collection, sepsis), and post‐discharge course without time limitations. We also collected hospital‐related costs for initial presentation and all subsequent related visits for patients seen at Sidra Medicine from 2018 to February 2024. This doesn't reflect patients' payments, as this is variable according to residency status.

### 2.4 Statistical analyses

Descriptive parameters were expressed as number (%), mean ± standard deviation (SD), or median (interquartile range [IQR]) as appropriate. Chi‐square test, Fisher's exact test, and Student's *t*‐test were used for statistical comparisons, as appropriate. Statistical significance was set at *p* < 0.05. A multivariate logistic regression analysis was planned, if appropriate, to calculate odds ratios (ORs) and 95% confidence intervals (CIs) for the association of different parameters with adnexal torsion.

## 3 RESULTS

Forty‐five patients were referred with magnet ingestion across the two institutions during the study period. All patients were admitted upon consultation. Of the 45 patients, 37 (82.2%) required intervention either immediately or after a period of observation, and only eight cases (20%) had spontaneous passage of the ingested magnet and did not require any intervention.

The male‐to‐female ratio was 1.66, which was the same in the intervention and non‐intervention groups (see Table 1). Overall, the majority of patients were in the pre‐school age of 3–5 years, followed by toddlers <2 years, and primary school age of 6–11 years (see Figure 1). Patients in the non‐intervention group were older than those in the intervention groups, with a median and range of 5.5 years (3–8 years) and 3 years (12–13 years), respectively (*p* < 0.001) (see Figure 2).

**Table 1:** Patient demographics of intervention and non‐intervention groups.

	Intervention (*n* = 37)	Non‐intervention (*n* = 8)	*p*‐value
Male	23 (62.16%)	5 (62.5%)	*p* > 0.05
Female	14 (37.84%)	3 (37.5%)	
Age (median and IQR) (years)	3 (1.62–4)	5.5 (4.94–6)	*p* > 0.05
Number of magnets (median and range)	5 (1–32)	2 (1–10)	0.005
Length of stay (median and IQR) (days)	6 (3.25–8.75)	1 (1–2.5)	0.005
Presence of symptoms	26 (70.27%)	2 (25%)	0.017
Initial management			
Observation	17 (45.95%)	8	0.005
Immediate intervention	20 (54.05%)	‐	

Abbreviation: IQR, interquartile range.

**Figure 1:**
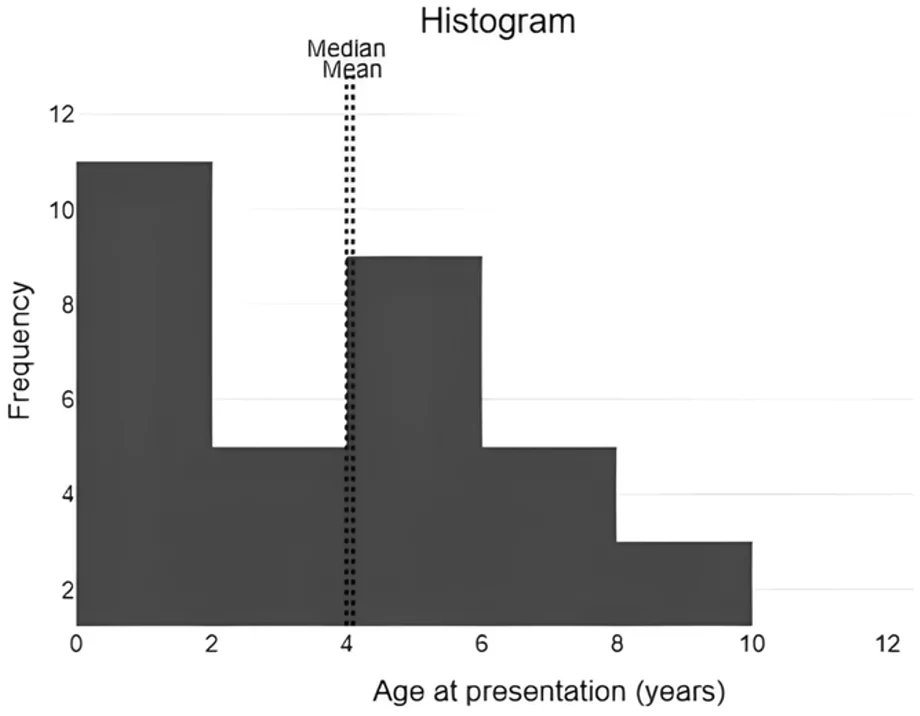
Age distribution of cases.

**Figure 2:**
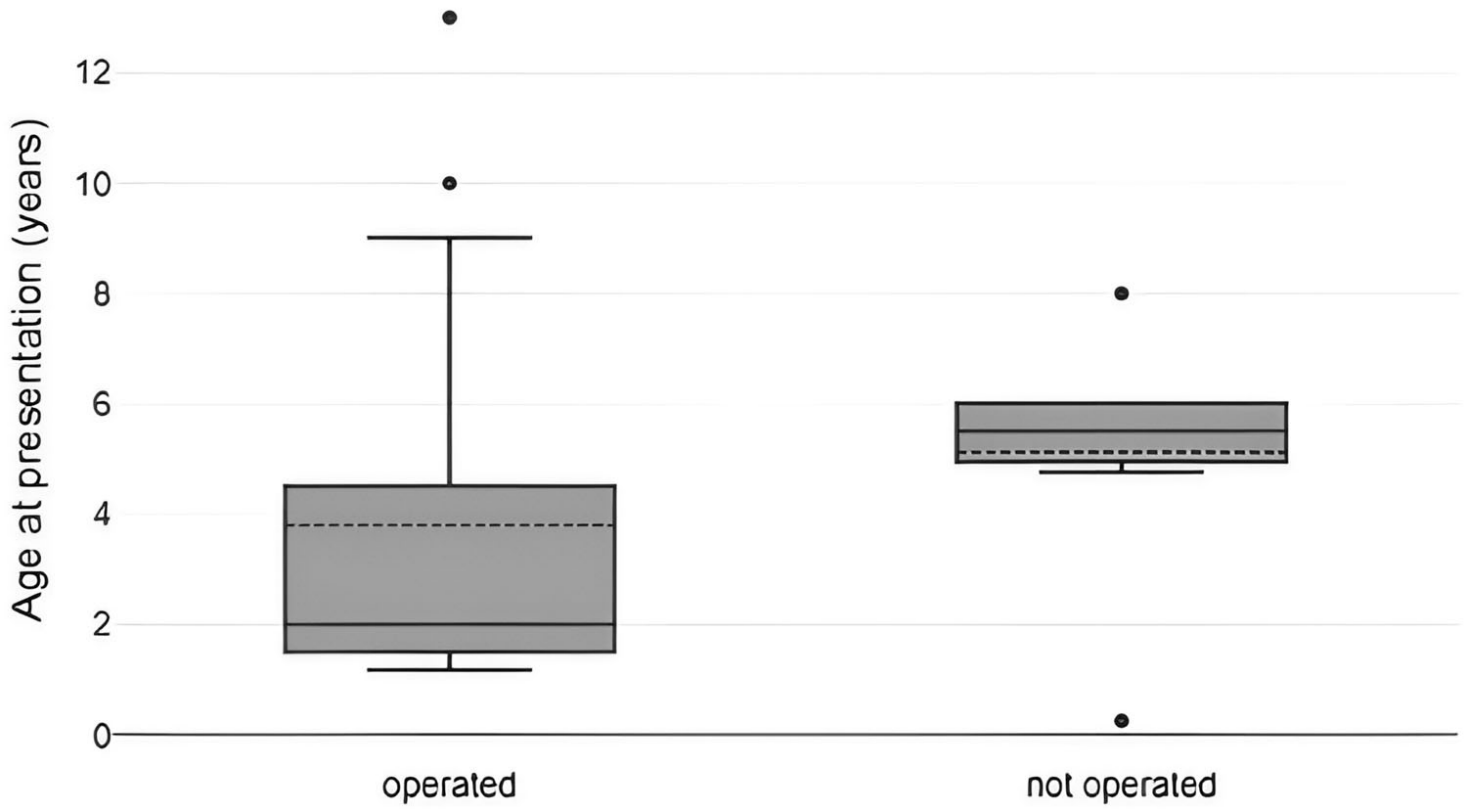
Box plot of the age of the intervention and non‐intervention group.

The number of patients managed initially by observation was 22 (55%). Of these 22 patients, only 8 were successful in the resolution confirmation, as verified by X‐ray (*n* = 7) or seen in the toilet (*n* = 1), and 1 was lost to follow‐up with no confirmation of passage nor intervention. The majority of patients who underwent the intervention were symptomatic (28/37, 78%). The most common symptoms in the intervention group were abdominal pain and nonbilious vomiting seen in 19/28 (67%). Four patients had associated abdominal distension (15%), and four reported constipation (15%). Other symptoms include nausea (2, 7%) and loose stools (2, 7%). Only two patients were symptomatic in the non‐intervention group (25%), both had abdominal pain (Figure 3). None of the patients in the present study experienced bilious vomiting.

**Figure 3:**
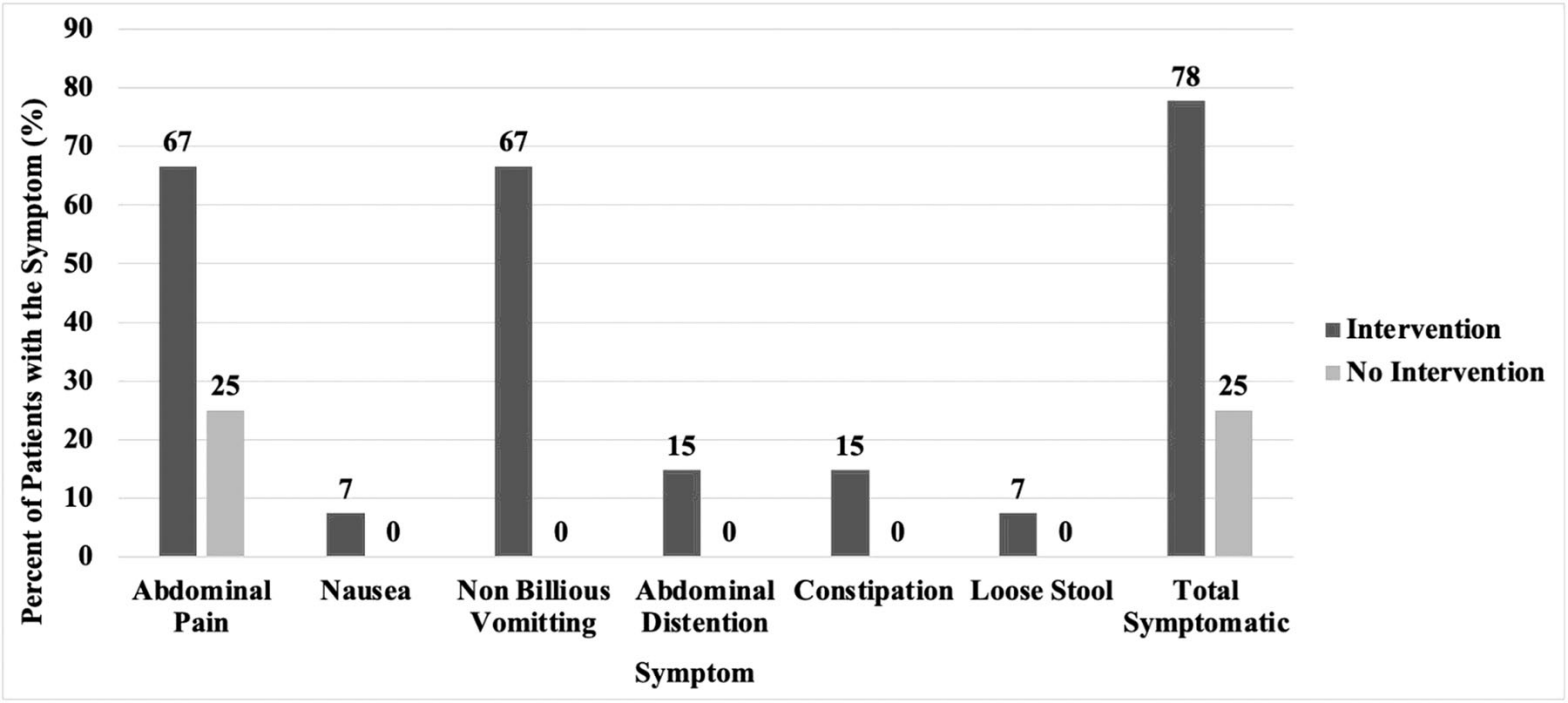
The percent of patients within the intervention and the non‐intervention group that were symptomatic. Information on presentation were missing for the five cases from HMC. HMC, Hamad Medical Corporation.

The most common indication for surgery was failure to pass the magnets (31.25%), followed by intestinal obstruction (25%). Four patients had peritonitis (12.5%), and two patients (6.25%) had frank perforation (Table 2). Of the 32 patients who underwent intervention, 21 (65.62%) required laparotomy, five (15.6%) required laparoscopy as the sole or combined modality (combined with endoscopy), and five (15.6%) were amenable to endoscopic removal only. One additional case (3.1%) required laryngoscopy and magnet removal from the hypopharynx. Complications post intervention were seen in 9/32 cases (28.1%). These include superficial wound infection (five cases), collection (three cases), adhesive bowel obstruction (one case), and incisional hernia (one case).

**Table 2:** Indication for intervention in patients with magnet ingestion (seen at Sidra Medicine, *n* = 32).

1. Failure to pass magnets	10 (31.25%)
2. Intestinal obstruction	8 (25%)
3. Other (magnets in hypopharynx or stomach)	6 (%)
4. Peritonitis in the absence of free air	4 (12.5%)
5. Frank perforation (free air)	2 (6.25%)
6. Not specified	2 (6.25%)

### 3.1 Factors predictive of intervention

Logistic regression was performed to assess the potential predictors of intervention. Age, presence of symptoms, and number of magnets were the three assessed factors. Age alone was not a predictor of intervention, with OR 1.03 (95% CI 0.93−1.14), *p* > 0.05. Likewise, the number of magnets was associated with OR 1.46 (0.96–2.22), which was not statistically significant (*p* > 0.05). The presence of symptoms was associated with OR 7.09 (1.23–40.75), which indicates an increase in the probability of surgery of 88% (*p* < 0.05).

### 3.2 Hospital‐related costs

Costs were summarized for 39 of 40 of the case that were cared for at Sidra Medicine from 2018 onwards. One patient had concurrent leukemia, and hospital costs involved treatment related to that, and was excluded. One patient was taken to OR for IV cannula insertion under sedation, with no further intervention for the magnet ingestion. The cost related to that was also excluded

The total cost of care for all 39 patients seen was USD 524,660.87 for a total of 229 hospital days with or without intervention. The median hospital‐related cost per patient was USD 7901.96 (IQR 3922.06, 13,699.88). For the patients admitted for observation only, median cost of care was USD 2056.92 per patient (IQR 1074.46, 2658.53), while the median costs for patients who have had any intervention was USD 10,564.37 per patient (IQR 6374.11, 14,899.4). Operating room (OR) related costs were a median of 3389.86 per patient (IQR 2167.26, 6278.49). Costs of medications was a median of USD 208.9 per patient (IQR 98.89, 398.17). The costs associated with emergency care was USD 458.08 (287.67, 841.12), while that associated with inpatient care was USD 7782.35 per patient (IQR 3739.58, 13,160.21). See Table 3 for details.

**Table 3:** Direct hospital‐level costs per patient in US Dollars.

Cost classification	No. of patients	Costs
Costs for observation	7	2056.92 (1074.46, 2658.53)
Costs for intervention (any)	32	10,564.37 (6374.11, 14,899.4)
OR charges		3389.86 (2167.26, 6278.49)
Encounter status		
Emergency	33	458.08 (287.67, 841.12)
Inpatient	39	7782.35 (3739.58, 13,160.21)
Medications	36	208.9 (98.89, 398.17)
Total costs per patient	39	7901.96 (3922.06, 13,699.88)

## 4 DISCUSSION

Ingestion of rare‐earth magnets in children can result in serious gastrointestinal injuries. Rare‐earth magnets exhibit a significantly higher magnetic force than ordinary magnets, and each magnet can attract more than five times the magnetic force of a normal magnet.^11^ When multiple rare‐earth magnets are swallowed or magnets are swallowed with other metallic foreign bodies, they can strongly attract each other, leading to compression of the intestinal wall, ischemia, necrosis, perforation, internal fistula, peritonitis, intestinal obstruction, and even death.^12^

The National Electronic Injury Surveillance System estimated 16,386 possible magnet ingestions among children aged 18 years in the United States from 2002 to 2011.^13^ In our case series, 45 patients presented with symptoms and 32 required interventions. This can be considered a national series for the country of Qatar with an estimated population of 2.8 million, of which about 260,000 are below 18 years of age.^14^

The ingestion of rare‐earth magnets is not limited to young children, and can extend into teenage years or even adulthood. There is a higher likelihood of autism, schizophrenia, or other psychiatric disorders in older age groups.^5^ In another published local series, magnet ingestion was witnessed by the caregiver in 13 patients (62%; 95% CI 38–82) and the median duration from the time of ingestion to presentation at the emergency department was 4 h (IQR 3–11), whereas in eight patients (38%; 18–62), the magnetic ingestion was unnoticed by the caregiver, and the duration from ingestion to presentation at the emergency department was uncertain.^12^

A systematic review of all magnetic foreign body ingestion cases revealed that in 119 of 149 cases, bowel injury was observed in the form of fistula, perforation, volvulus, mesenteric vascular injury, and internal herniation.^5^ In our case series, 75% of the patients who underwent intervention had similar complications.

Prompt diagnosis and management is essential to avoid complications related to magnet ingestion. Imaging in the form of X‐Ray in two views is diagnostic in most cases. Some reports urged the importance of imaging neck to pelvis to avoid missing magnets in multiple sites.^15^ Scouting for dislodged magnets in the pharynx and the cervical esophagus in the assessment phase will aid in deciding on the type of intervention for retrieval. Current NASPAGHAN guidelines indicate the need for urgent removal of multiple magnets when ingested.^11^ This includes the asymptomatic patients when the location is amenable to endoscopic retrieval (esophagogastroduodenoscopy or colonoscopy).

In a large multicenter cohort study, Neville et al. found that younger age and number of magnets were associated with the need for intervention.^6^ In our case series, 80% of patients with rare earth magnet ingestion required intervention in the form of endoscopy or surgery, and the number of magnets and the presence of symptoms were predictive of the need for surgery. *Symptomatic patients had 7.1 higher odds (88% probability) of needing intervention compared to non‐symptomatic patients*.

Taking strict and drastic measures to limit the access of children to these magnets would have the greatest impact on their safety. Several studies have highlighted the relationship between the policy on magnet sale and its ingestion by children and associated injuries. Product recall has been shown to be an effective mechanism for reducing harmful downstream effects. This is done by limiting the production/availability of new products, destroying existing products in the market, or limiting their availability, and educating consumers on the hazards to raise awareness. In 2014, the US Consumer Product Safety Commission (CPSC) approved federal safety standards and issued a recall of rare‐earth magnets from the market. This was overruled in 2016, and sales were re‐established. There was a clear drop in the rate of injuries during the ban, followed by an increase in the incidence of magnet ingestion injuries highlighted in several US‐based tertiary care centers.^16^ In 2013, Canada implemented a similar mandatory nationwide product recall. Prior to the recall, magnet ingestion incidence in one tertiary care center was 18 per 100,000 ED visits, this drops to less than 2 per 100,000 ED visits 2 years after the recall.^17^ Moreover, magnet extraction procedures dropped from 14 (41%) to 4 (33%) whereas surgical interventions dropped from 6 (18%) to 1 (8.3%).^17^

In 2022, the US Consumer Product Safety Commisin (CPSC) issued a notice restricting and regulating the marketing of “hazardous magnets,” defines as those with flux index of 50 kg^2^ mm^2^ or more.^18^ Sales of magnet toys are not restricted nor limited in Qatar or neighboring GCC countries. A recent multi‐national study including centers from Qatar, UAE, Saudi, Turkey and Tunisia highlighted that this is a regional hazard with 189 cases reviewed, 37% requiring intervention (endoscopy or surgery), and 20% requiring emergency surgical intervention upon admission.^19^

Hospital‐related costs in magnet ingestion in children is not well studied in children. An Australian study of intentional ingestion of foreign bodies in a psychiatric hospital setting showed an average cost per hospital day of the equivalent of USD 1638.73, and estimated of total cost of USD 1115,320.30 for their cohort of 35 patients (3294.86 per patient).^20^ Their cohort had repeated admissions in many of their patients, explaining the higher costs observed.^20^ Considering the differences in healthcare systems and reliance on insurance in the US based hospitals versus government supported health care in Qatar, one could still see the financial and resource burden this exerts on the healthcare system. An intervention banning such toys would result in much needed reduction in morbidity as well as saving of hospital costs. Our study highlighted that the majority of expense are associated with inpatient care. A potential future change would be to optimize resource utilization and triaging selected patients (asymptomatic, and older) to close outpatient observation. Symptomatic patients who according to our study, are more likely to need intervention, and younger patients, who are less likely to communicate symptoms clearly, are best admitted for close observation.

This study was limited by its retrospective nature, with missing or insufficient data. Data prior to 2018 were especially difficult to acquire, and the number of cases might have been under estimated. Subjective data related to socioeconomic factors that might have predisposed to magnet ingestion, such as bigger family size, the presence of a working mother, and socio‐economic status, were not available to review for the patients seen. Also, the economic burden incurred by magnet ingestion was not reflected in the data.

## 5 CONCLUSION

Magnet ingestion poses a significant health hazard in children and predisposes them to significant morbidity and even mortality. Prompt diagnosis with liberal X‐rays and a low threshold for intervention is the most reliable way to avoid complications. In addition to morbidity on patients, this specific issue carries a sizeable financial burden on hospitals and healthcare sector. The safety of close outpatient observation in selected patients needs to be explored before suggesting it as means to help reduce the burden on hospital resources. Further reforms should be considered including investing in public education in schools and malls as well as social media campaigns, as well as exerting a ban on the toys in the country.

## CONFLICT OF INTEREST STATEMENT

The authors declare no conflicts of interest.

## Data Availability

The datasets used and analyzed during the current study are available from the corresponding author on reasonable request.
